# Cellular death, reactive oxygen species (ROS) and diabetic complications

**DOI:** 10.1038/s41419-017-0135-z

**Published:** 2018-01-25

**Authors:** Caroline Maria Oliveira Volpe, Pedro Henrique Villar-Delfino, Paula Martins Ferreira dos Anjos, José Augusto Nogueira-Machado

**Affiliations:** Núcleo de Pós-Graduação e Pesquisa, Hospital Santa Casa de Belo Horizonte, Rua Domingos Vieira 590, Santa Efigênia, Belo Horizonte, MG30150-240 Brazil

## Abstract

Chronic or intermittent hyperglycemia is associated with the development of diabetic complications. Several signaling pathways can be altered by having hyperglycemia in different tissues, producing oxidative stress, the formation of advanced glycation end products (AGEs), as well as the secretion of the pro-inflammatory cytokines and cellular death (pathological autophagy and/or apoptosis). However, the signaling pathways that are directly triggered by hyperglycemia appear to have a pivotal role in diabetic complications due to the production of reactive oxygen species (ROS), oxidative stress, and cellular death. The present review will discuss the role of cellular death in diabetic complications, and it will suggest the cause and the consequences between the hyperglycemia-induced signaling pathways and cell death. The signaling pathways discussed in this review are to be described step-by-step, together with their respective inhibitors. They involve diacylglycerol, the activation of protein kinase C (PKC) and NADPH-oxidase system, and the consequent production of ROS. This was initially entitled the “dangerous metabolic route in diabetes”. The historical usages and the recent advancement of new drugs in controlling possible therapeutical targets have been highlighted, in order to evaluate the evolution of knowledge in this sensitive area. It has recently been shown that the metabolic responses to stimuli (i.e., hyperglycemia) involve an integrated network of signaling pathways, in order to define the exact responses. Certain new drugs have been experimentally tested—or suggested and proposed—for their ability to modulate the possible biochemical therapeutical targets for the downregulation of retinopathy, nephropathy, neuropathy, heart disease, angiogenesis, oxidative stress, and cellular death. The aim of this study was to critically and didactically evaluate the exact steps of these signaling pathways and hence mark the indicated sites for the actions of such drugs and their possible consequences. This review will emphasize, besides others, the therapeutical targets for controlling the signaling pathways, when aimed at the downregulation of ROS generation, oxidative stress, and, consequently, cellular death—with all of these conditions being a problem in diabetes.

## Introduction

Diabetes mellitus (DM) is considered a metabolic and inflammatory disease that affects more than 170 million people around the world. Its increase worldwide is epidemic. Despite a new generation of medications and the advances in clinical treatments, the prevalence of diabetes has risen dramatically. Diabetes is characterized by hyperglycemia, and its control only slowly hinders the progression of the disease’s complications, without stopping them. Hyperglycemia triggers several metabolic signaling pathways that lead to inflammation, cytokines secretion, cell death, and, consequently to diabetic complications. These are represented by inflammation in the vessels and/or in the nerves, such as in nephropathy, retinopathy, and cardiovascular diseases. Diabetes seems to be more complex than it appears. It seems to involve a network of metabolic signaling and at this time science does not know how to control this signaling.

Hyperglycemia activates a particular metabolic route that involves diacylglycerol (DAG)—protein kinase C (PKC)—and NADPH-oxidase—culminating in ROS, previously having been suggested as the “dangerous metabolic route in diabetes”^1^. This particular signaling pathway has received attention for the control of angiogenesis, oxidative stress with decreased ROS production, and cellular death. It is nowadays accepted that ROS is induced by hyperglycemia in diabetic patients through mitochondrial respiratory chain enzymes, xanthine oxidases, lipoxygenases, cyclooxygenases, nitric oxide synthases, and peroxidases^2–5^.

It would appear that the current therapeutical options that are available for the treatment of diabet only serve to slow the progression of diabetic complications. New biomarkers and therapies are urgently needed in order to control the secretion of cytokines, the production of AGEs, vascular inflammatory complications, and the modulation of cellular death. Several diverse steps along the metabolic signaling route hyperglycemia-induced in diabetes could potentially be considered new therapeutical targets.

Glycemic controls, in conjunction with modulation of PKC and/or NADPH-oxidase, downregulated the pro-inflammatory cytokines, leading to a reduced amount of ROS, and, consequently, decreased cellular death. This could be an option for controlling these diabetic complications. However, this is not an easy task.

**Fig. 1:**
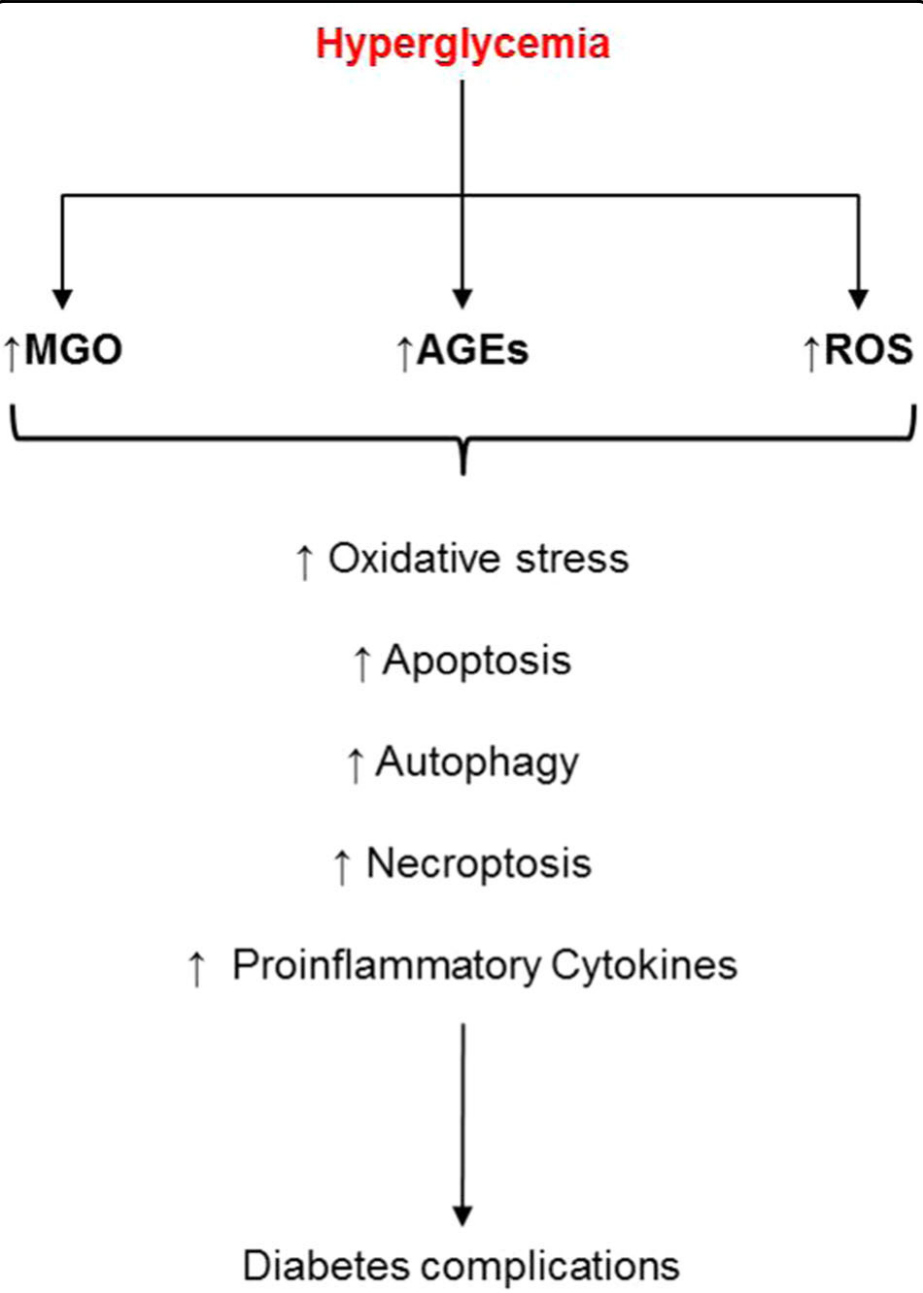
Oxidative stress and cell death: necroptosis, a programmed necrosis of inflammatory cell, and apoptosis can be induced in diabetes by AGEs, ROS, and MGO, leading to diabetes complications (retinopathy, age-related macular etc.) MGO = methylglyoxal, AGEs = advanced glycation end products, ROS = reactive oxygen species.

Hyperglycemia is the trigger for the activation of several signaling pathways, and it represents a condition in which the cells become susceptible to necroptosis, apoptosis, and/or necrosis^6^. Both Type 1 and 2 diabetes mellitus (T1D and T2D) are metabolic disorders, apparently with distinct mechanisms, but with a significant loss of mass insulin-producing β-cells, due to cellular death^7^. The death cell (apoptosis) in diabetes is activated by inflammatory cytokines and involves caspase, resulting in pancreatic β-cell death^8^. It has been demonstrated that methylglyoxal (MGO), a reactive dicarbonyl metabolite of glucose, together with ROS, have induced apoptosis in human umbilical vein endothelial cells (HUVECs), but this can be downregulated by caspase inhibitors^9^. In addition, cellular death has also been reported in diabetic retinopathy, in age-related macular degeneration, and in programmed necrosis of the inflammatory cells, with all of them resulting from the action of AGEs, ROS, and MGO (Fig. 1)^6,9^.

Apparently, diabetic complications have an increment of ROS and oxidative stress, leading to cellular death by several mechanisms, and, then finally, tissular damage. In different models, the diabetic complications that are induced by hyperglycemia appear to be due to an imbalance between the oxidizing species (ROS), leading to oxidative stress and cellular death^10–14^. Therefore, the downregulation of ROS generation may have a pivotal role in controlling diabetic complications.

This review will emphasize the therapeutical targets needed for controlling the signaling pathways of DAG—PKC—NADPH-oxidase, step-by-step, as well as for the downregulation of ROS generation, oxidative stress, and, consequently, cellular death. This disclosure will allow for the proposal of new deep studies and new targets.

## Cellular death and diabetes complications

The increased ROS generation leads to oxidative stress, inducing several cellular changes. Acute or chronic high glucose in diabetes increases the production of ROS and activates apoptosis in the β-cells^15^. Both apoptosis and necroptosis have important roles in the progression of diabetic complications and they may culminate in tissue injuries in the heart, retina, kidneys, and nervous system^16,17^.

**Fig. 2:**
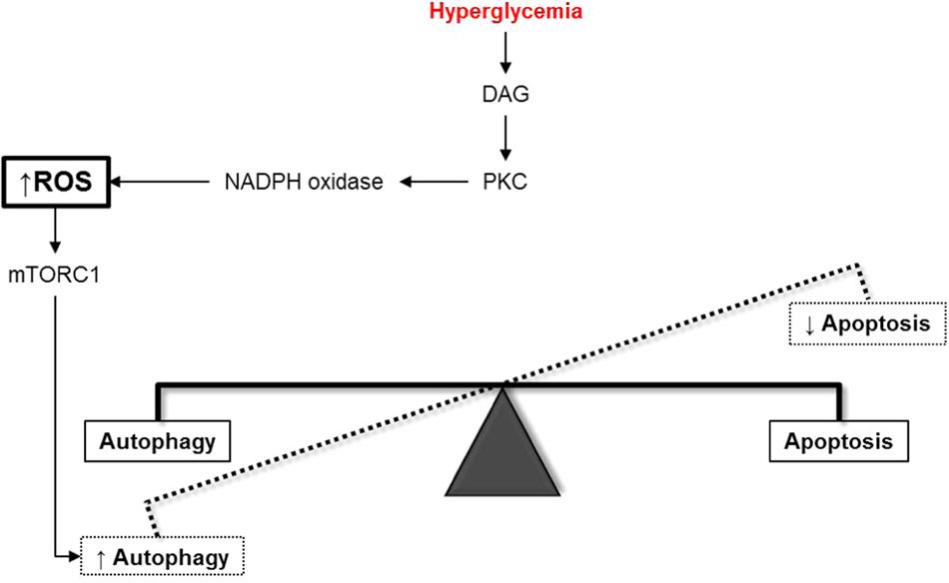
Balance between autophagy and apoptosis: apoptosis is downregulated by autophagy The activation of mTORC1 depends on PKC, which is activated by DAG. Thus, PKC in conjunction with ROS and mTORC1 pathway are associated in the control of cellular autophagy. Akt/mammalian target of rapamycin complex 1 = mTORC1, ROS reactive oxygen species, PKC = protein kinase C, DAG = diacylglycerol.

Autophagy, necroptosis, and apoptosis are the kinds of cellular death with different functions. Autophagy is a catabolic and homeostatic process for the lysosomal degradation of damaged organelles, protein aggregates, and recycling materials. Autophagy is a critical step in tissular damage. The imbalance between autophagy and apoptosis may establish the progression of diabetes complications^17^. Apoptosis is downregulated by autophagy (Fig. 2). It has been reported that the role of the mammalian target of rapamycin complex 1 (mTORC1) is a downstream of the AKT pathway in apoptosis^13^. The activation of mTORC1 depends upon PKC. Thus, PKC, in conjunction with the mTORC1 pathway, is both associated with the control of cellular autophagy. An inefficient activation of mTORC1 may be reflected in the suppression of autophagy^13^.

**Fig. 3:**
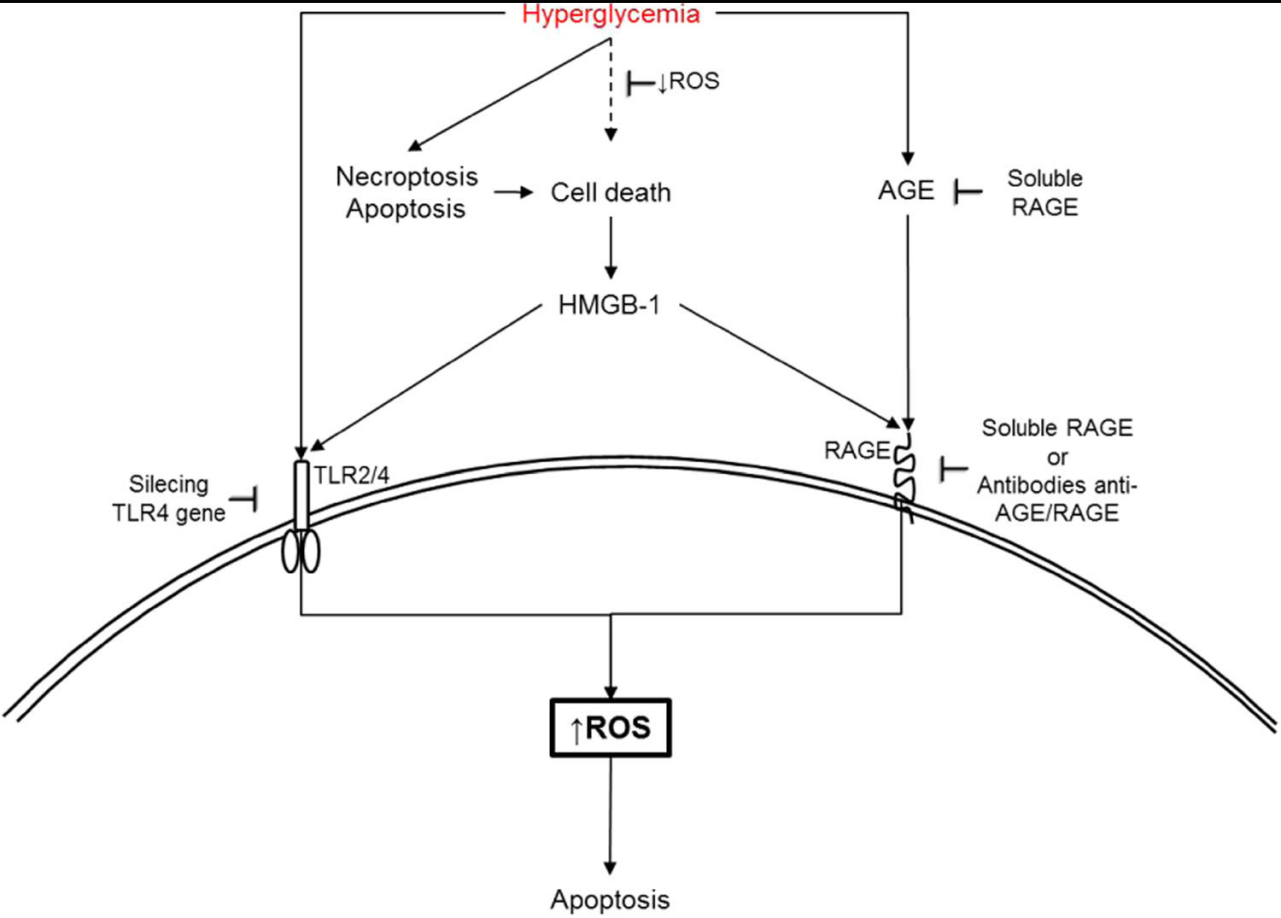
Targets for modulation of cell death: some points may act as modulators of cell death depending on the kind of activation Apoptosis induced by TLR activation has been suggested to be downregulated by silencing of TLR4 gene in experimental model or using soluble RAGE and/or specific antibodies against RAGE. Cell death is a source of HMGB-1, an activator of RAGE. TLR TLR-Toll-like receptors, RAGE receptor for advanced glycation end products.

Besides hyperglycemia and ROS, apoptosis can be triggered by an activation of Toll-Like Receptor 4 (TLR4). The upregulation of the TLR4 expression has been demonstrated in diabetic mice, and the silencing of the TLR4 gene could control cardiac apoptosis in diabetes. TLR4 deficiencies have inhibited ROS production and NADPH oxidase activities. This might lead to the downregulation of oxidative stress and inhibition of caspase 3 in cardiomyocytes^14^. It has been reported that AGEs, lipoprotein-associated phospholipase A2, adiponectin, tumor necrosis factor-alpha (TNF-α), chemokines, interleukin (IL)-1β, IL-6, IL-8, IL-18 and high mobility group box-1 (HMGB-1) are all critical inflammatory mediators of diabetic complications. HMGB-1 itself can activate receptor for advanced glycation end products (RAGE), and the TLR2 and TLR4, resulting in NF-kB activation that leads to upregulation of the pro-inflammatory cytokines, the adhesion molecules, and the angiogenic mediators. All of these aggravate the inflammatory processes in diabetes, leading to cellular death^1,18–24^. The role of AGE and TLR can be better visualized in Fig. 3.

Autophagy is usually a protective process however, there are some extreme conditions in which it is injurious to tissues and induces cellular death by apoptosis or necrosis. Both autophagy and apoptosis need to act in balance^11,13^. An autophagy dysfunction process has been reported in several pathological conditions, such as diabetes, cancer, and neurodegenerative diseases^10,12,25,26^. An impaired autophagy may be induced by high glucose in diabetes, and, hence, it may induce an accumulation of unfolded protein and dysfunctional organelles inside the cells. All of this is associated with the role of autophagy in the pathophysiology of diabetes complications^27^.

Endoplasmatic reticulum (ER) is responsible for protein folding and maturation, besides other functions. A dysfunction of ER may produce unfolded protein responses leading to ER stress. ER stress itself has induced apoptosis in diabetic rat tissues. ROS activate the formation of ER stress, leading to apoptosis. The ER stress sensor protein kinase RNA (PKR)-like ER kinase (PERK) appears to be the sensor that is responsible for the activation of apoptosis in diabetic cardiomyocytes. Myocytes that are depleted of PERK, but not of inositol-requiring enzyme 1 (IRE1) and/or activation transcription 6 (ATF6), have protected cells against hyperglycemia-induced apoptosis^28^. Yuan et al.^29^have suggested that the dysfunction and the apoptosis of the β-cells, that are induced by the free fatty acids (FFAs), depend upon the NOX2- derived ROS. NOX4 is a subunit of the NADPH-oxidase complex and the activation of the TGF-β1/Smad2 signaling pathway is suggested to be the main reason responsible for the ROS generation, and, consequently, apoptosis in vascular endothelial cells^30^.

The use of LY2109761, a selective inhibitor of TGF-β1/Smad2, diphenyliodonium (DPI), and/or astragaloside IV (AST IV is a monomer located in an extract of astragaloside) decreased the NOX4 expression, the level of ROS, and the TGF-β1/Smad2 expression, suggesting that inhibitions of the NOX4 and TGF-β1/Smad2 pathways downregulate apoptosis in HUVECs^30^. In a similar approach, the compound L6H9 decreased the generation of ROS, cytokine secretions, and the level of apoptosis in the cardiomyocytes of STZ-induced diabetic mice, as well as controlled their diabetic heart complications^31^.

Another group of experiments with promising results was performed when using injections of recombinant TNF-related apoptosis-inducing ligand (TRAIL) in streptozotocin diabetes-induced rats. The results demonstrated that TRAIL protected vascular lesion, controlled ROS production and apoptosis, as well as inhibited NADPH-oxidase complex, together with the AKT/PKB anti-apoptotic signaling pathways. However, it also increased nitric oxide (NO), due to the activation of endothelial nitric oxide synthase (eNOS)^32^. Glutaredoxin-1 (GRX-1) is an antioxidant cytosolic enzyme that activates the eNOS/NO system producing NO. It inhibits the JNK and NFk-B pathways, downregulating both oxidative stress and apoptosis. GRX-1 has protected hyperglycemia-induced coronary damage^33^.

It has also been reported that lithium treatments of PC12 cell line decrease the generation of ROS, while at the same time inhibiting caspase 3, the phosphorylation of JNK, the mitogen-activated protein kinase (P38MAPK) pathway, as well as hyperglycemia-induced apoptosis. The authors have suggested that lithium could be an efficient tool for modulating the development of neuropathy^34^. The compound diallyl trisulfide (DATS) has been able to inhibit the generation of ROS, the translocation of NFk-B, the activation of caspase 3, and NADPH-oxidase, besides interfering with the JNK and C-Jun signaling pathways, as well as downregulating apoptosis in the cardiomyocytes of streptozotocin-treated diabetic rats. DATS has been suggested as a potential therapy for diabetic cardiomyopathy^35^. Hyperglycemia-induced oxidative stress and increased ROS production are associated with cellular death of cardiomyocytes, endothelial cells, tubular epithelial cells, tubular atrophy basement membranes, neuronal cells, and renal failure.

## Inhibition of ROS production for controlling cellular death in diabetes

**Fig. 4:**
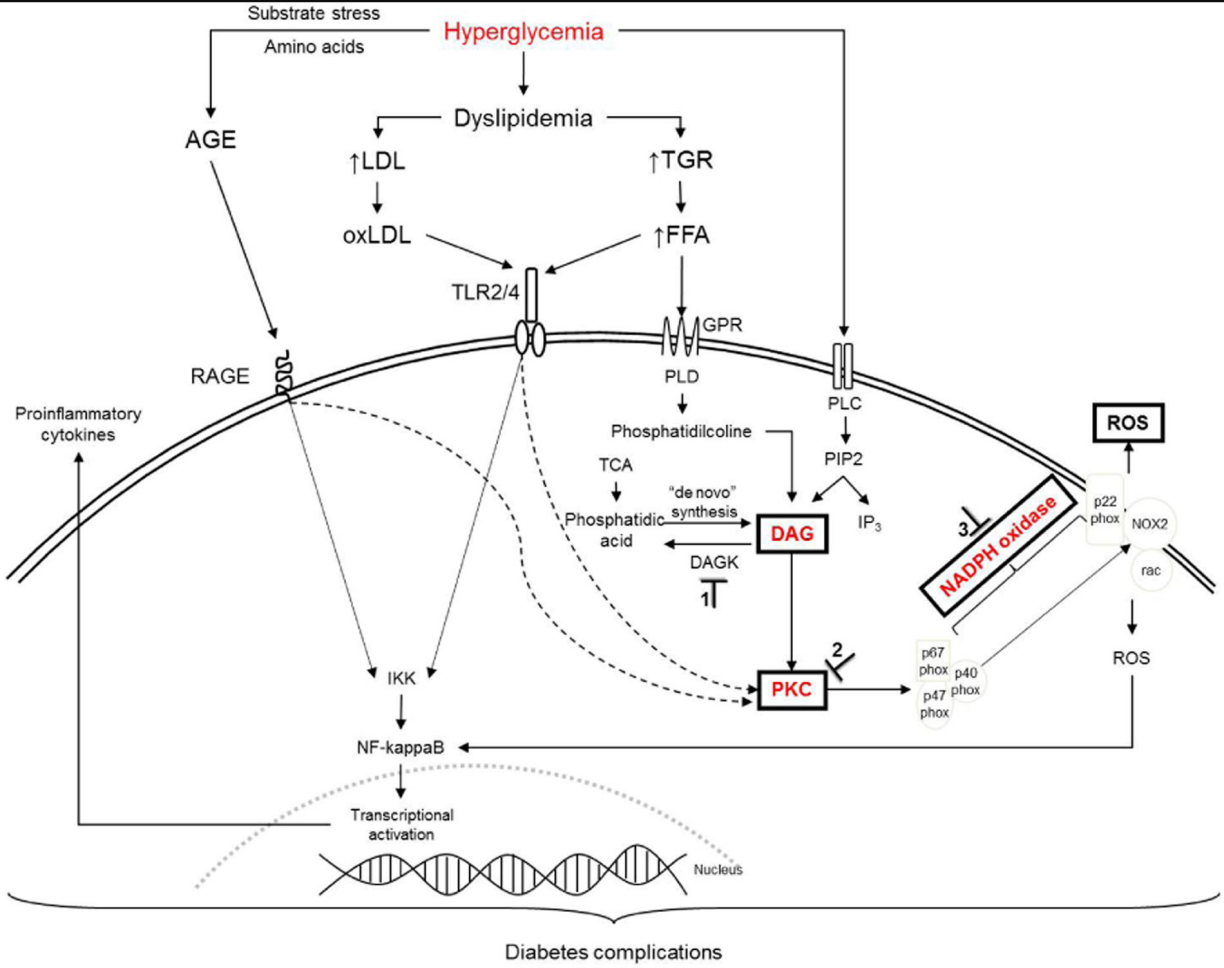
Proposed signaling pathways from hyperglycemia to ROS production and cytokine release leading to diabetes complications Hyperglycemia increases diacylglycerol (DAG) content by the activation of phospholipase C or D, which activates protein kinase C (PKC). PKC activates NADPH oxidase. NADPH oxidase complex consists of the cytosolic components p47phox, p67phox, p40phox, and a low-molecular-weight G-protein, Rac 1 or Rac 2, and the membrane-associated NOX2 and p22phox. Activation of the enzyme complex requires translocation of the cytosolic components to the plasma membrane, and their association to NOX2, produces reactive oxygen species (ROS). AGE-RAGE, FFA-TLR, and oxLDL-TLR activate downstream IKK (IkappaB kinases) pathways, and PKC. IKK phosphorylates IkappaB (inhibitor of kappa light chain gene enhancer in B cells), leading to translocation of the transcription factor NF-kappaB to the nucleus to control the expression of pro-inflammatory cytokines. **⊥** points of inhibition. 1 Inhibition of diacylglerol kinase; 2 Inhibition of PKC; 3 Inhibition of NADPH oxidase. AGE advanced glycation end products, DAG diacylglycerol, DAGK diacylicerol kinase, FFA free fatty acid, GPR G-protein-coupled receptors, IKK IkappaB kinases, IP3 inositol trisphosphate, LDL low density lipoprotein, NF-kappaB nuclear factor, oxLDL oxidized low density lipoprotein, PIP2 phosphatidylinositol-4,5-bisphosphate, PKC protein kinase C, PLC phospholipase, PLD phospholipase D, RAGE receptor for advanced glycation end products, ROS reactive oxygen species, TCA triacylglycerol, TLR Toll-like receptor.

There are several studies on new compounds that are able to control the production of ROS along the signaling pathways of DAG-PKC-NADPH-oxidase that are activated by hyperglycemia (Fig. 4).

### ROS inhibition by diacylglycerol inhibitors: is this a good option?

High blood glucose levels in diabetes activate DAG formation, a physiological activator of PKC. This phosphorylates the subunits of NADPH-oxidase, leading to the production of ROS. The substrate stress that is represented by hyperglycemia in diabetes reacts with the amino acids and proteins to form AGEs, which interact with RAGE on the cell surfaces, to produce the pro-inflammatory cytokines. Both ROS and AGE are associated with oxidative stress, inflammation, and cellular death. When blocking an activator of PKC (DAG), the metabolic signaling cascade is interrupted and ROS production is inhibited. An inhibition of the DAG formation is one of the options to control this signaling pathway.

DAG is formed from distinctive metabolic routes. Briefly, phospholipase D (PLD) and phospholipase C (PLC) are activated by hyperglycemia in diabetes. Both PLD and PLC act on phosphatidylcholine and phosphatidylinositol bisphosphate (PIP2), respectively, leading to a formation of DAG. In a similar way, phosphohydrolase 1 and phosphohydrolase 2 (PAP1, PAP2) may induce “de novo” synthesis of DAG from phosphatidic acid (PA). DAG may also be formed from PA by an action of diacylglycerol kinase (DAGK). Because of its biochemical complexity and its diverse sources, DAG does not seem to be a good therapeutical target. However, DAG is the physiological activator of PKC and this is associated with oxidative stress. In the vascular cells from a diabetic rat, it has been reported that after a hyperglycemia peak DAG remained in an increased state for at least 3 weeks^36–39^. This may mean that high DAG levels could sustain the activation of PKC, and, consequently, the activation of NADPH-oxidase, in order to produce ROS (“dangerous metabolic route in diabetes”)^1^.

There are only a few reports in the literature about the use of diacylglycerol inhibitors, as being a therapeutical resource for ROS inhibition and/or cellular death for controlling diabetic complications. Most of the suggestions are related to an inhibition of DAGK. However, these DAGK inhibitors were mainly reported for tumors and immunotherapy, not for diabetes. Recent studies have revealed that DAGK isozymes have important roles in several signaling transduction pathways, conducting growth factor/cytokine-dependent cell proliferation and motility, seizure activities, immune responses, cardiovascular responses, and insulin receptor-mediated glucose metabolism^40^. More than ten isoforms of DAGK have been reported and their inhibitions have been associated with therapeutical targets for several pathologies^41^.

The compound R59949, a diacylglycerol kinase inhibitor, acts on inducible nitric oxide synthase (iNOS) production and IL-1β-secretion. This clearly suggests a very intricate signaling network that involves DAG/DAGK, NO, and NADPH-oxidase. Other DAGK inhibitors, for instance, the compound R59022, induced cancer cell death in vitro and in vivo^42^, but no suggestions have been found for its use in diabetes. In diabetes, theoretically, the inhibition of PLC, PLD, or even DAGK could increase the levels of DAG by de novo synthesis, thus, inducing much worse oxidative stress . A DAG formation from PA is a reversible chemical reaction and a DAGK inhibition could also enhance the de novo synthesis of DAG formations. Because of these scenarios, a direct DAG inhibition is not the best therapeutical target option for diabetes.

### PKC inhibitors and ROS/cellular death inhibition

PKC represents a family of kinases composed of isoforms dependent on DAG and Ca^2+^ activation. The families are divided into α, βI, βII, and γ dependents of DAG and Ca^2+^. A further family is divided into δ, ε, η, and θ. These are Ca^2+^ independent, however, they are DAG dependent. A third family is both Ca^2+^ and DAG independent. The domains of C1 and C2 bind DAG and Ca^2+^, respectively. Hyperglycemia activates PLC/PLD to form DAG, and, consequently, this activates PKC. PKC is involved with the activation and the synthesis of MAPK, vascular endothelial growth factor (VEGF), and transforming growth factor β (TGF-β), with NO being released via an activation of iNOS. PKC also has a pivotal role in the vascular dysfunction of hyperglycemia-induced retinal vascular permeability and in the expression of VEGF. On inhibiting PKC, and, consequently, the production of ROS, the death cells and pro-inflammatory cytokines will be downregulated.

The involvement of PKC in several signaling pathways represents one of the most important therapeutical targets for controlling inflammation in diabetic complications. NADPH-oxidase is activated by PKC to produce ROS. Some medications that act as a PKC inhibitor have been proposed and they have been tested in experimental studies and clinical trials, in order to control angiogenesis, and possibly ROS and cellular death, in diabetic retinopathy. Experiments using these kinds of compounds could be evaluated for cellular death (apoptosis and autophagy). Among them, the medications of Ruboxistaurin, PKC-412 (staurosporine), and PKC-412A (N-benzoyl-staurosporine) were all tested in trials by Eli Lily & Co., as oral medications, in order to inhibit PKC-β and to control diabetic macular oedema, retinopathy, neuropathy, and other angiopathies^43,44^. It has been demonstrated that Ruboxistaurin showed a renoprotective role, by the downregulation of the TGF-β1/Smad pathway, as well as the GRAP (Grb2-Related Adaptor Protein) expressions in streptozotocin rat diabetes^45^. It has been suggested that Ruboxistaurin should be used for blocking the VEGF expression and reducing the visual losses in diabetic retinopathy. This treatment should only be used before there are any lesions in the eyes and not to control an injury that has already taken place^46^. Retinopathy leads to expressive inflammatory processes in angiogenesis, in macular oedema, and in lesions of the retina. This may suggest the involvement of cellular death. Maleimide Derivative 3 is a compound similar to Ruboxistaurin with the highest of binding affinities and this might also be used in the treatment of diabetic retinopathy^47^. A diabetic rat that was treated with the PKC-βII inhibitor showed diminished expressions of TNF-α, ICAM-1 (Intercellular Adhesion Molecule 1), NFk-B-Bp65, and Caspase 3, besides protecting hepatic ischemia and reperfusion^48^.

Some products have been proposed to be registered as patents, but most of them were neither tested experimentally nor tested in pharmaceutical trials. Pyrazolopyrimidines have been proposed as tyrosine kinase inhibitors for VEGF Receptor 2, MAPK, ERK (Extracellular Signal-Regulated Protein Kinase), CDKs (Cyclin-Dependent Protein Kinases) and RAF1. The main effects of pyrazolopyrimidines would be acting as an anti-angiogenic agent, in order to control diabetic complications^49^. It is reasonable to suggest that the control of angiogenic responses in hyperglycemia-induced ocular pathology is associated with several other active mechanisms, such as the inhibition of the cytokine secretions, cellular death, and the inhibition of NFk-B, among others.

Currently, there are reports of more than a hundred new PKC inhibitor drugs that are under evaluation. N-pyrimidin-4-yl-3-amino-pyrrolo [3,4-C] pyrazole derivatives working as PKC inhibitor, being selective for PKCβI, PKCβII, and PKCα^50^. They have been suggested as a PKC-based drug for the treatment of diabetic complications—but no clinical trials are ongoing.

### NADPH-oxidase inhibitors and ROS/cellular death inhibitions

It is to be expected that an inhibition of PKC and NADPH-oxidase leads to similar results, particularly in terms of the inhibitions regarding the production of inflammatory mediators. The NADPH-oxidase enzymatic complex is composed of sub-units, namely, p22phox, and NOX2 (membrane-associated); p47phox; p67phox; p40phox; and an activation of the small GTPase Rac (cytosolic units). The NOX family can be expressed in vasculature terms as NOX1, NOX2, NOX3, NOX4, and NOX5. It is well known that, NOX family is responsible for the ROS production in several pathologies, such as diabetes^51–55^.

Other consequences are the expression of MMP2, MMP9, VEGF, RAGE, together with an increased blood pressure, due to the activation of the Renin—Angiotensin—Aldosterone System (RAAS)^56–58^. It is to be expected that the use of the PKC and NADPH-oxidase inhibitors could modulate the expression of these inflammatory mediators, leading to the downregulation of diabetic complications. NADPH-oxidase and PKC form the main therapeutical targets, in order to control the metabolic consequences of hyperglycemia in diabetes.

Several drugs have been tested as inhibitors of NADPH-oxidase. Rutin has protected the endothelial dysfunction, inhibiting the NOX4 response to oxidative stress, and the ROS-sensitive NLRP3 signaling pathway that is under hyperglycemia stress, in both in vivo and in vitro situations^59^. The compound GKT137831 is already under clinical trials and it controls oxidative stress in diabetic vasculopathy by inhibiting the NOX1 and NOX4 sub-units of NADPH-oxidase^60^. A highly selective compound of NOX4 that is specified as GLX351322 has been proposed as a therapeutical strategy for Type 2 diabetes mellitus^60^. It prevents ROS generation and cellular death^61^. Diabetic hyperglycemia-induced cardiomyopathy in rats was downregulated by GYY4137, through a slow release of the chemical compound H2S. It also reduced the expression of NOX4 in cardiac fibroblasts^62^.

Medications that are already in use in clinical practices, such as metformin, rapamycin, and statin, may act as NADPH-oxidase inhibitors, downregulating the generation of ROS, and, consequently, oxidative stress^62–64^. Other compounds have been studied, such as triptolides, apocynin, diphenyleneiodonium (DPI), triazolopyrimidine and its derivatives, GKT36901, spironolactone (antagonist of the aldosterone receptor), and the metabolites of losartan (EXP3179). All of these have been reported as inhibitors of NADPH-oxidase^65–76^. However, most of the evidence comes from in vitro experiments, or from animal models, when evaluating the effects of these substances as being suitable NADPH-oxidase inhibitors. Clinical trials using substances for controlling the complications of diabetes have not yet been realized. Experiments are needed in order to study the effects of these inhibitors on apoptosis, necroptosis, and autophagy. These compounds, which are able to control oxidative stress and ROS production, could be efficient in downregulate cellular death.

## Conclusion

This review has evaluated cellular death and it has been based on several metabolic signaling mechanisms emphasizing the control of the pathways of DAG—PKC—NADPH-oxidase, mainly in relation to downregulation of ROS generation. The increase of ROS production and cellular death are associated. The modulation of various steps of signaling that are induced by hyperglycemia could be potential therapeutical targets in diabetes, for blocking ROS generation, cytokine secretions, and cellular death. It is known that besides its osmotic effects on cells, hyperglycemia activates DAG formation, together with an activation of PKC and NADPH-oxidase, which leads to the production of ROS and oxidative stress in diabetes. In diabetes, these inflammatory reactions are mediated by a pathological cellular death (apoptosis and autophagy) and they may be aggravated by the action of AGEs, due to the afterward consequences of an interaction with the respective receptor (RAGE) (Figs. 1–3). All of this leads to chronic low-grade inflammation that is responsible for diabetes complications. Any attempt to control these problems could modulate some of the consequences of hyperglycemia. PKC and NADPH-oxidase have been suggested as being the best targets, in order to control hyperglycemia-induced oxidative stress.

It has become necessary to deeply evaluate the therapeutical targets that have been suggested and the possible consequences that may occur on the metabolic networks.

This present review has suggested a signaling pathway important in diabetes that, in conjunction with other metabolic matters, could downregulate oxidative stress, the pro-inflammatory cytokine secretions, as well as cellular death. In some situations, hyperglycemia may induce cellular death and lead to complications in diabetes and tissue injuries. It is believed that diabetic complications are the consequences of persistent hyperglycemia-induced low-grade inflammation and cellular death.

It is believed that diabetic complications are the consequences of cellular death and a persistent hyperglycemia-induced low-grade inflammation.

It is regrettable to conclude that, despite the great advances in diabetic therapy that have already been obtained science is still far from a definitive solution for controlling the development of diabetic complications. Certainly, the solutions will pass by regardless, without a complete understanding of those signaling pathways that are affected by hyperglycemia in diabetes. The control of ROS production, mediators of inflammation and pathological apoptosis, and autophagy is mandatory. The assumptions of the researchers are that the same inhibitors used for controlling oxidative stress and angiogenesis in diabetic complications could also be used for controlling pathological cellular death. Thus, the arsenal of compounds that have been suggested for controlling diabetes complications could also be used for controlling pathological cellular death in diabetes and other pathologies.

In conclusion, all of the scientific advances that have already been obtained are insufficient in avoiding the endemic profile of diabetic diseases and the evolution of its complications.
